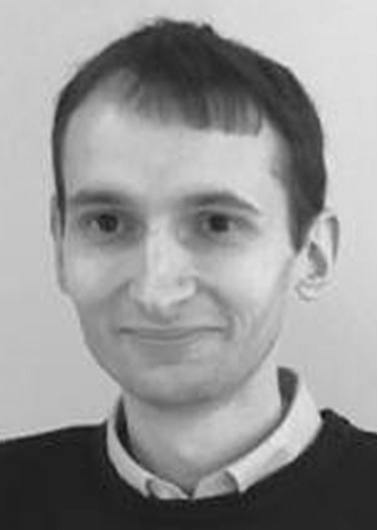# Chiral Catenanes and Rotaxanes: Fundamentals and Emerging Applications

**DOI:** 10.1002/chem.201704149

**Published:** 2017-11-30

**Authors:** Nicholas H. Evans

**Affiliations:** ^1^ Department of Chemistry Lancaster University Lancaster LA1 4YB UK

**Keywords:** catalysis, catenanes, chirality, host–guest recognition, rotaxanes

## Abstract

Molecular chirality provides a key challenge in host–guest recognition and other related chemical applications such as asymmetric catalysis. For a molecule to act as an efficient enantioselective receptor, it requires multi‐point interactions between host and chiral guest, which may be achieved by an appropriate chiral 3D scaffold. As a consequence of their interlocked structure, catenanes and rotaxanes may present such a 3D scaffold, and can be chiral by inclusion of a classical chiral element and/or as a consequence of the mechanical bond. This Minireview presents illustrative examples of chiral [2]catenanes and [2]rotaxanes, and discusses where these molecules have been used in chemical applications such as chiral host–guest recognition and asymmetric catalysis.

## 1 Introduction

**Figure 1:**
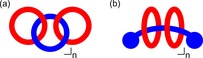
Schematic representations of: a) [*n*+2]catenane, and b) [*n*+2]rotaxane structures.

Any undergraduate chemistry student will be familiar with the concept of chirality, and specifically that chiral molecules are non‐superimposable on their mirror image. Many processes in the natural world rely on the ability to recognize chiral molecules, and this, in part at least, inspires chemists to study chiral host–guest recognition and related themes such as asymmetric catalysis.^1^ It is generally accepted that a 3D arrangement of at least three interactions (one of which needs to be stereochemically dependent) must exist between a chiral host and its chiral guest to achieve chiral recognition.^2^ As a consequence of their interlocked structures, catenanes^3^, ^4^ (molecules consisting of two or more interlocked macrocyclic rings, Figure 1 a) and rotaxanes^5^ (molecules consisting of stoppered axle(s) components threaded through one or more macrocyclic rings, Figure 1 b) could form the basis of useful 3D scaffolds for chiral hosts.

It is now well‐established that template synthesis provides a versatile route to interlocked molecules,^6^ relying on the preorganization of components prior to final covalent bond formation to trap the interlocked species.^7^ Classical templating strategies using metal cations,^8^ π–π stacking^9^ and hydrogen bonding^10^ have been supplemented by more recent work on anionic,^11^ radical–radical^12^ and halogen bond^13^templation. Although synthetic methodology is still being developed, research is increasingly being focused on exploiting mechanically interlocked molecules in chemical applications,^14^ such as host‐guest recognition^15^ or catalysis.^16^ Some researchers have made use of the 3D geometries created by interlocked molecular structures to achieve guest selectivity, and some have exploited the stimulus‐controlled motion of interlocked components to make examples of molecular machines^17^ which were celebrated by the 2016 Nobel Prize in Chemistry.^18^

A catenane or rotaxane may be made chiral by the inclusion of a classical chiral motif. Alternatively, chirality may arise as a consequence of the mechanical bond. Although examples of chiral interlocked molecules have been known for some time (as evidenced by previous reviews),^19^, ^20^ it is perhaps only now that this research field has begun to genuinely flourish, as significant progress is not only made in their preparation, but also in the application of these topologically and stereochemically exotic species. This Minireview sets out to provide an informed overview of examples of chiral catenanes and rotaxanes, before detailing their use in emerging chemical applications. As a Minireview, this article is not designed to be an exhaustive collection of every chiral catenane or rotaxane, and instead a range of illustrative examples will be presented, focusing principally on chiral examples of [2]catenanes and [2]rotaxanes, and those that exhibit useful chemical application. For progress in the related class of molecular knots, interested readers are referred to an excellent review that has just been published.^21^

## 2 Chirality Arising from Classical Chiral Elements

**Figure 2:**
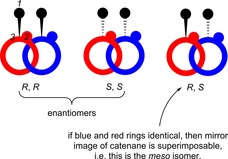
Schematic representation of possible stereoisomers of a homocircuit [2]catenane, where each ring contains a single stereogenic centre (the numbers in italics are to illustrate CIP priority assignment).

A straightforward way to create a chiral catenane or rotaxane is by incorporating a classical chiral element, such as a chiral centre, axis or plane into at least one of the components that make up the interlocked molecule. However, care is needed in certain cases. For example, for a homocircuit [2]catenane (a catenane where the two interlocked rings are identical), if one ring has a stereocentre with an *R* configuration and the other an *S*, then the catenane is achiral (it is the *meso* diastereomer of the *R*,*R* and *S*,*S* enantiomeric pair, see Figure 2).

### 2.1 Catenanes that possess classical chiral elements

**Figure 3:**
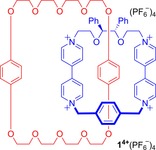
Stoddart's catenane **1** ^4+^(PF_6_^−^)_4_ containing a tetra‐pyridinium macrocycle with two stereogenic centres.

**Figure 4:**
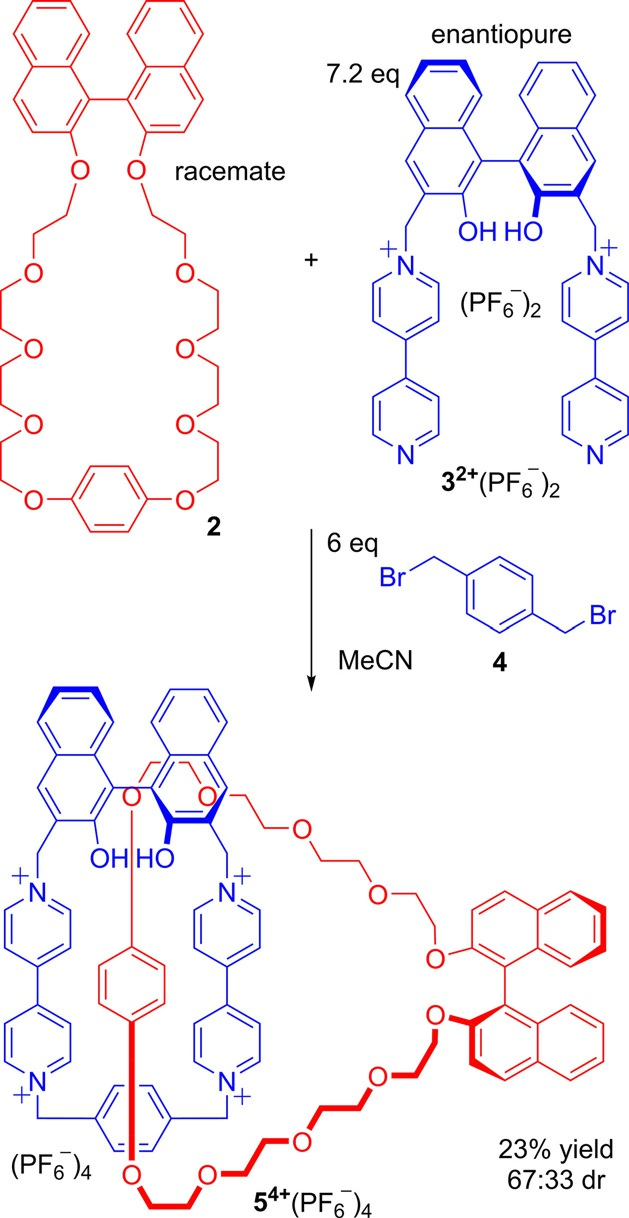
Diastereoselective synthesis of Stoddart's catenane **5** ^4+^(PF_6_^−^)_4_ containing two rings, both possessing axial chirality.

**Figure 5:**
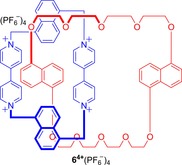
Stoddart's catenane **6** ^4+^(PF_6_^−^)_4_ containing two rings, both possessing planar chirality.

Catenanes possessing chirality arising from a chiral centre,^22^ axis^23^ and plane^24^ may be exemplified by work from the group of Stoddart (Figures 3, 4 and 5). All were prepared by clipping shut a tetra‐pyridinium macrocycle around an electron rich aromatic motif of a crown ether macrocycle—self‐assembled by charge assisted π–π donor‐acceptor templation.

In cases of [2]catenanes, in which both rings possess chiral elements, researchers have sometimes observed evidence of diastereoselectivity in synthesis. For example, when racemic binaphthyl crown ether **2** and enantiopure (*R*)‐binapthyl bis‐pyridinium precursor **3** were used, the *R*,*R* and *S*,*R* stereoisomers of the catenane were obtained in a diastereomeric ratio of 67:33 in favour of *R*,*R* (Figure 4).^23^

For catenane **6** ^4+^(PF_6_^−^)_4_ (Figure 5), both rings may possess elements of planar chirality, when the pair of binaphthyl spacers in each ring are staggered with respect to one other. In solution, twelve stereoisomers of this catenane are possible (six diastereomers, each existing as pairs of enantiomers), many of which are in exchange with one another through low energy conformational and co‐conformational processes. Although this catenane exhibits complex dynamic stereochemical behaviour in solution, the researchers reported that crystallization afforded enantiopure single crystals of the *SS*, *SS* enantiomer.^24^

### 2.2 Rotaxanes that possess classical chiral elements

**Figure 6:**
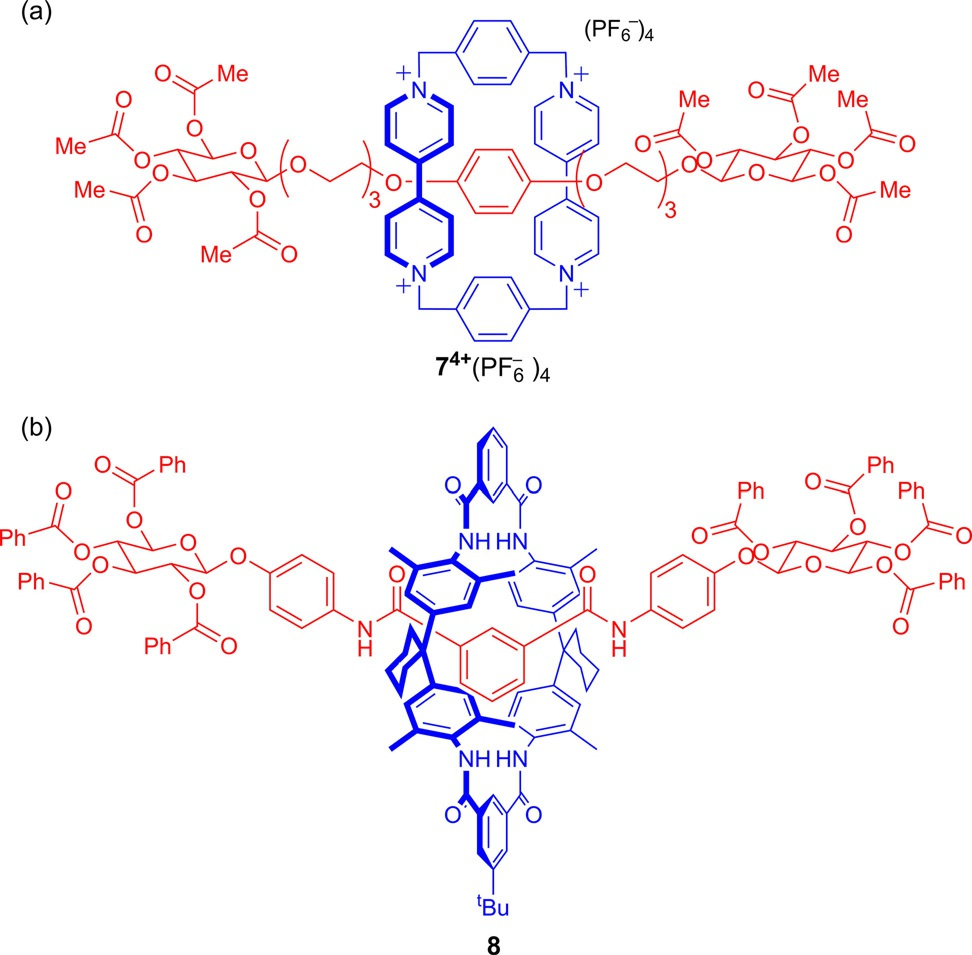
Vögtle's glucose stoppered rotaxanes **7** ^4+^(PF_6_^−^)_4_ and **8**, prepared by: a) donor–acceptor and b) hydrogen‐bond templating.

Amongst early work, Vögtle and co‐workers prepared charge assisted π–π donor–acceptor rotaxanes possessing chiral tetraacetyl glucose stoppers (such as **7** ^4+^(PF_6_^−^) in Figure 6 a).^25^ In addition, the same group prepared examples of hydrogen bond templated rotaxanes stoppered with tetrabenzoyl glucose units (such as **8** in Figure 6 b).^26^ In the latter case, the rotaxanes exhibited amplified circular dichromism (CD) compared to the free axle.

**Figure 7:**
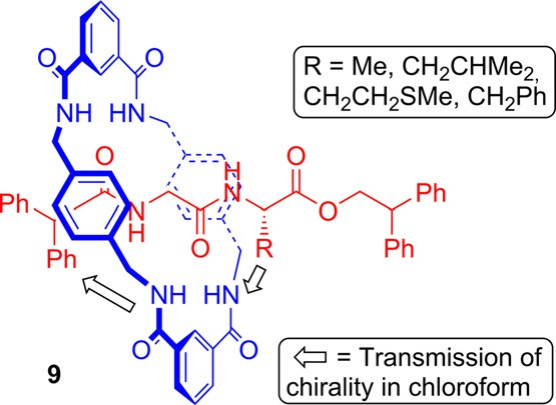
Leigh's amino acid containing rotaxanes **9** that demonstrate solvent, temperature and chiral substituent dependent ICD.

Subsequently, Leigh and co‐workers have prepared a series of hydrogen bond templated rotaxanes incorporating l‐amino acids (Figure 7).^27^ In chloroform, the rotaxanes **9** exhibit CD signals, but the corresponding non‐interlocked chiral axles do not. Detailed investigations revealed that chirality is transmitted from the chiral centre on the axle via the interlocked macrocycle to the C‐terminal stopper of the rotaxane. Further, this induced circular dichroism (ICD) may be modulated by varying solvent, temperature or size of the chiral substituent. For example, switching the solvent to hydrogen bond competitive methanol leads to a dramatic reduction in CD signal for any given rotaxane as the macrocycle can freely rotate and hence does not communicate the chiral information efficiently.

**Figure 8:**
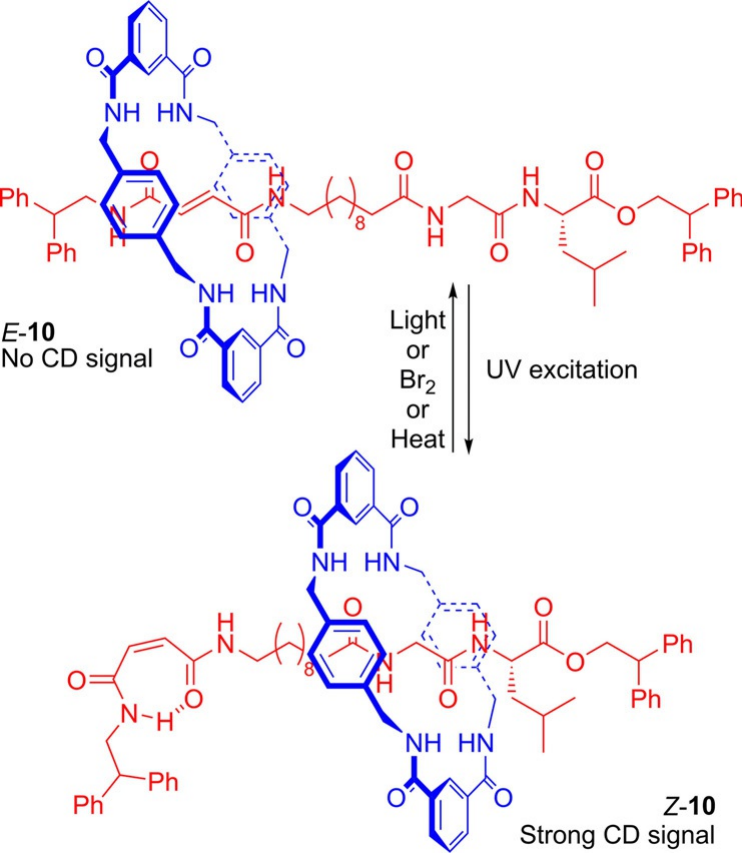
Leigh's bistable molecular shuttle **10** that demonstrates chiroptical switching.

In follow‐up work, the same group reported on the use of light‐controlled *E*/*Z* isomerism in a related rotaxane **10** to create a chiroptical switch (Figure 8).^28^ When the fumaramide C=C is *trans*, then the macrocycle preferentially resides over this functional group. Isomerization of the double bond to *cis* geometry, causes the macrocycle to shuttle to the glycine‐leucine station, which, incorporating a chiral substituent, allows for the switching on of a chiral optical response (that is the appearance of a signal in the CD spectrum).^29^

## 3 Chirality Arising as a Consequence of the Mechanical Bond

Exciting opportunities are possible because of the mechanical bond, for chirality may arise in catenanes and rotaxanes even when the interlocked components are themselves achiral. This may be described as “mechanical chirality”, a term that has recently been defined specifically by Bruns and Stoddart as “*a non‐classical form of chirality resulting from the spatial arrangements of component parts connected by mechanical bonds*”.^30^, ^31^

**Figure 9:**
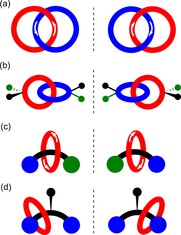
Schematic representations of enantiomers of [2]catenanes and [2]rotaxanes arising as a consequence of the mechanical bond: a) [2]catenane consisting of two directional rings; b) [2]catenane consisting of facially unsymmetric rings; c) [2]rotaxane consisting of directional ring and axle components and d) [2]rotaxanes consisting of a ring trapped on one side of what would be a prochiral centre of the non‐interlocked axle component.

Mechanical chirality may arise in [2]catenanes from directionality in both rings (Figure 9 a) or from facially unsymmetric rings (Figure 9 b). Catenanes possessing directionality in both rings may be described as topologically chiral. Mechanical chirality originates in [2]rotaxanes from both the axle and the macrocycle being directional (Figure 9 c) or when a macrocycle is trapped on one side of what would be a prochiral centre of the non‐interlocked axle component—in a manner with parallels to atroposiomerism (Figure 9 d).

### 3.1 Topologically chiral catenanes

**Figure 10:**
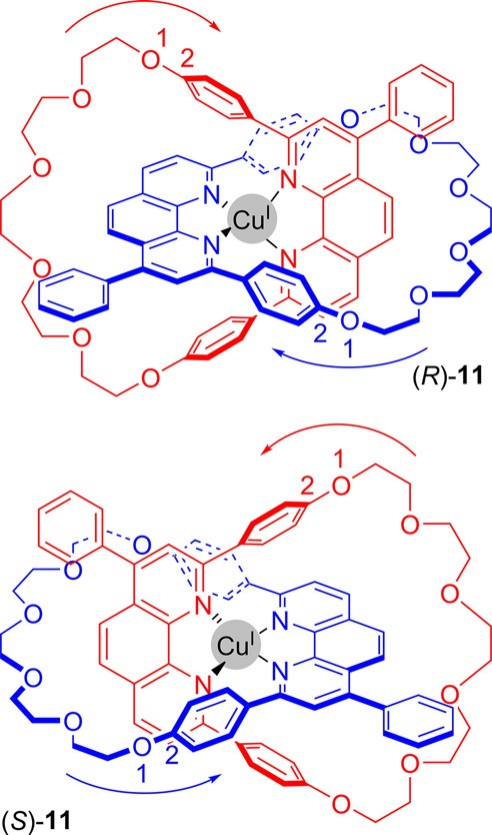
Enantiomers of Sauvage's first topologically chiral catenane **11**. The stereochemical labels *R* and *S* are assigned thus: in the direction of an arrow pointing from the highest priority atom (labelled 1) to its highest priority neighbour (labelled 2), interlocked rings that are disposed in a clockwise manner are *R*, those in an anticlockwise manner are *S*.

The first topologically chiral catenane was prepared by Mitchell and Sauvage by use of Cu^I^ cation templation.^32^ Inclusion of a phenyl substituent at position 4 of the phenanthroline group of both rings leads to the creation of enantiomers (Figure 10). The chirality of catenane **11** (which was prepared as a racemate) was confirmed by use of Pirkle's reagent (*S*‐(+)‐2,2,2‐trifluoro‐1‐(*p*‐anthryl)ethanol) and careful ^1^H NMR spectroscopic analysis. Subsequently, partial separation of the enantiomers was achieved by HPLC to allow for recording of mirror image CD spectra (in collaboration with Okamoto and co‐workers).^33^

**Figure 11:**
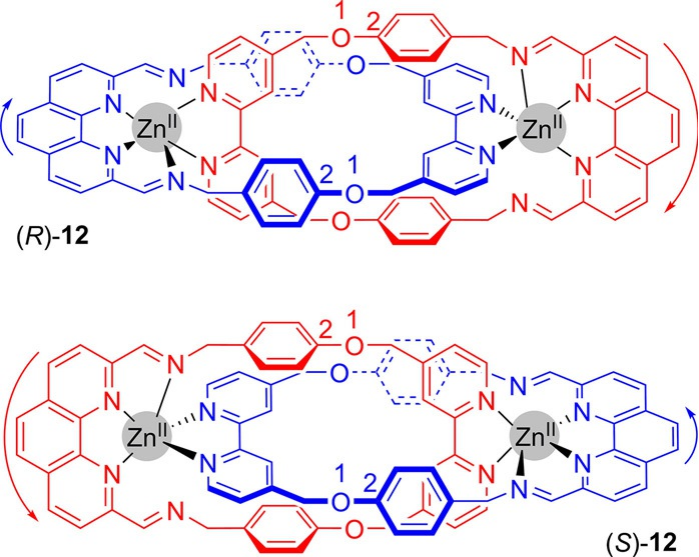
Enantiomers of Charbonnière and Tranolsi's catenane **12**. The numbers and arrows are to illustrate how stereochemical labels are determined.

An alternative source of directionality in metal templated mechanically chiral catenanes has been illustrated in an example of a bimetallic [2]catenane (Figure 11).^34^ In the solid state, crystal structures reveal that only one of the imine nitrogen atoms is coordinating to the Zn^II^ cation, thus generating directionality in each of the interlocked rings. However, in solution, catenane **12** is fluxional, with cleavage and formation of coordination bonds occurring. Such processes are forbidden when considering topology, and so in solution (at least) this catenane cannot be considered topologically chiral.

### 3.2 Facially unsymmetric chiral catenanes

**Figure 12:**
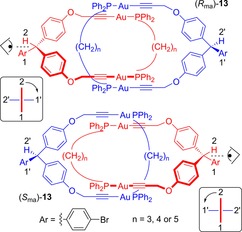
Puddephatt's catenanes, where chirality arises from facially unsymmetrical rings. The stereochemical labels *R*_ma_ and *S*_ma_ are assigned as one would assign *R* and *S* to allenes.

The first facially unsymmetric chiral catenanes were reported by Puddephatt in 2002 (Figure 12).^35^, ^36^ A set of [2]catenanes **13** were prepared using aurophilic templation. Chirality arises from the orientation of the bromo‐aromatic substituents, in a manner similar to how chirality arises in appropriately substituted allenes. Bruns and Stoddart have therefore proposed the use of the term “mechanically axial chirality” and the stereochemical labels *R*_ma_ and *S*_ma_ to describe the chirality in such catenanes.^30^

**Figure 13:**
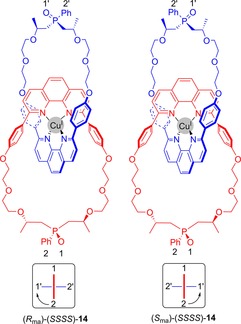
Diastereomers of Marinetti's facially unsymmetric mechanically chiral catenane **14**. The numbers and arrows are to illustrate how the *R*_ma_ and *S*_ma_ stereochemical labels are determined.

A Cu^I^‐templated catenane has been prepared by Marinetti and co‐workers that also exhibits facially unsymmetric mechanical chirality (Figure 13).^37^ In this case, facial dissymmetry arises from incorporation of phosphine oxides in each ring. In addition to the mechanical chirality there are stereogenic carbon atoms. By use of an enantiopure macrocycle to generate the catenane, the resulting diastereomers of demetallated catenane **14** were separable by preparative HPLC to give optically pure diastereomers.

### 3.3 Mechanically planar chiral rotaxanes

**Figure 14:**
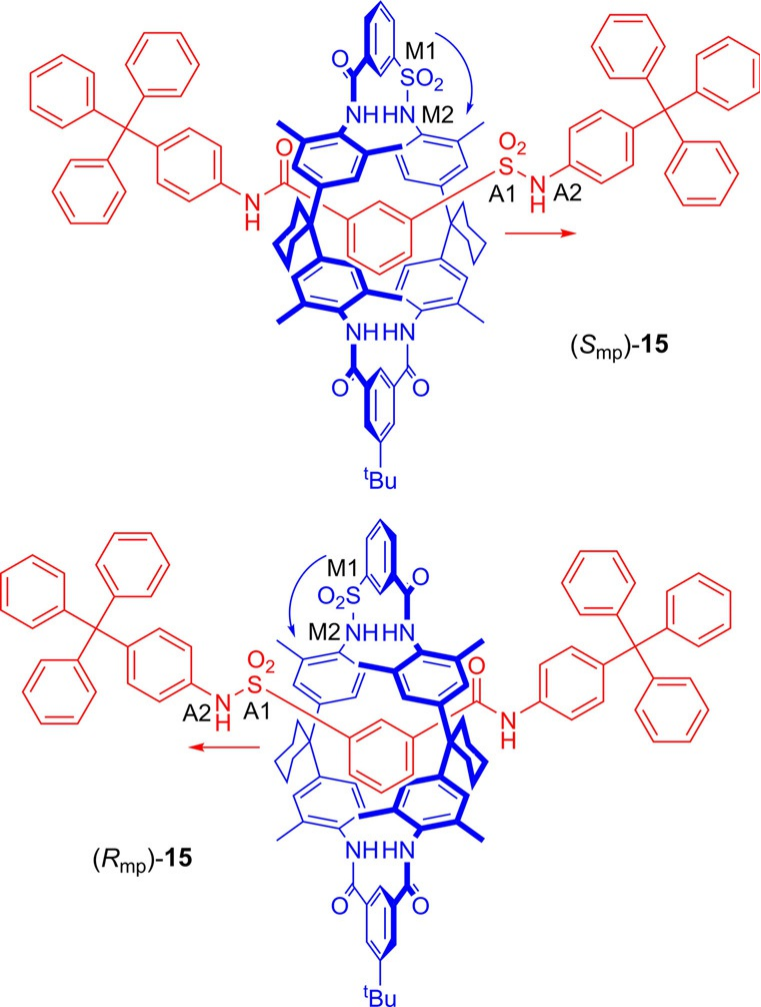
Enantiomers of Vögtle's mechanically planar chiral [2]rotaxane **15**. The stereochemical labels *R*_mp_ and *S*_mp_ are assigned thus: View the rotaxane along its axle from the highest priority atom in the axle (A1) to that atom's highest priority neighbour (A2). If in the macrocycle the highest priority neighbour (M2) is disposed clockwise from the highest priority atom (M1), then the stereochemical label is *R*_mp_; if it is disposed anticlockwise then the label is *S*_mp_.

In 1997, Vögtle and Okamoto reported upon the preparation and HPLC resolution of an amide‐sulfonamide [2]rotaxane, that is chiral by consisting of directional axle and macrocyclic components (Figure 14).^38^ Although the researchers successfully separated the enantiomers of rotaxane **15** and recorded mirror‐image CD spectra, they were unable to assign the absolute configuration of the two enantiomeric samples.

The authors of this paper used the term “cycloenantiomeric” to describe the chirality of **15**, but it has since been proposed that the term “mechanically planar chiral” is possibly a more appropriate descriptor.^39^ Goldup has also suggested a nomenclature for describing the enantiomers of a mechanically planar chiral rotaxane, as illustrated for rotaxane **15** in Figure 14.

**Figure 15:**
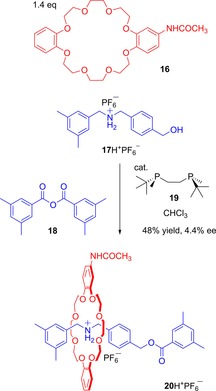
Takata's catalytic asymmetric synthesis of a mechanically planar chiral rotaxane (only one rotaxane enantiomer depicted, note the configuration of the major enantiomer was not established).

The key synthetic challenge in exploiting mechanically planar chiral rotaxanes is to prepare enantiopure examples on a preparative scale. An illustration of the challenge involved is reflected by Takata's report on attempts to achieve catalytic asymmetric synthesis of a planar chiral rotaxane consisting of a substituted 18‐dibenzocrown‐6 macrocycle and secondary ammonium salt axle (Figure 15).^40^, ^41^ The rotaxanes were prepared by an acylative end‐capping using a chiral bisphosphine catalyst. Unfortunately, the maximum observed enantiomer excess was only 4.4 %.

**Figure 16:**
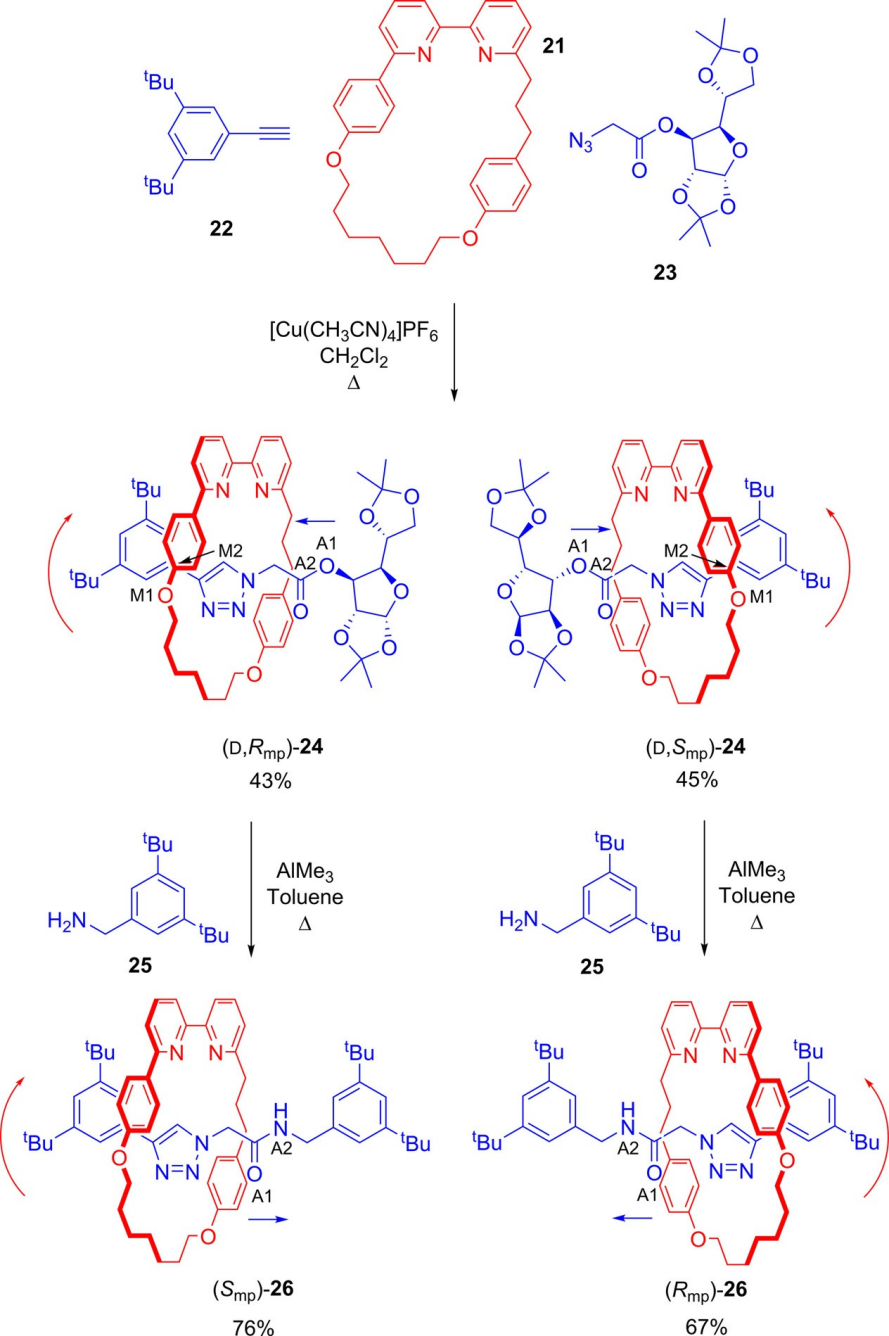
Goldup's preparation of enantiopure mechanically planar chiral rotaxanes. The labels (A1, etc) and arrows are to illustrate how the *R*_mp_ and *S*_mp_ stereochemical labels are determined.

A critical breakthrough in accessing enantiopure mechanically chiral rotaxanes was achieved in the elegant work of Bordoli and Goldup (Figure 16).^39^, ^42^ Using a CuAAC “click”‐active metal template synthesis,^43^ they constructed rotaxane **24** from directional macrocycle **21** and two half threads—achiral alkyne **22** and the other enantiopure azide **23**. The diastereomers of **24** were formed in an essentially 1:1 ratio, but crucially they could be separated by standard flash chromatography. Substitution of the chiral stopper for an achiral one then generated enantiopure samples of the enantiomers of the mechanically planar chiral rotaxane **26**. By obtaining crystal structures of the two diastereomers of rotaxane **24**, the researchers were also able to assign the absolute configurations of both diastereomers of **24** and the resulting enantiopure samples of mechanically chiral rotaxane **26**.

### 3.4 Point mechanical chiral rotaxanes

**Figure 17:**
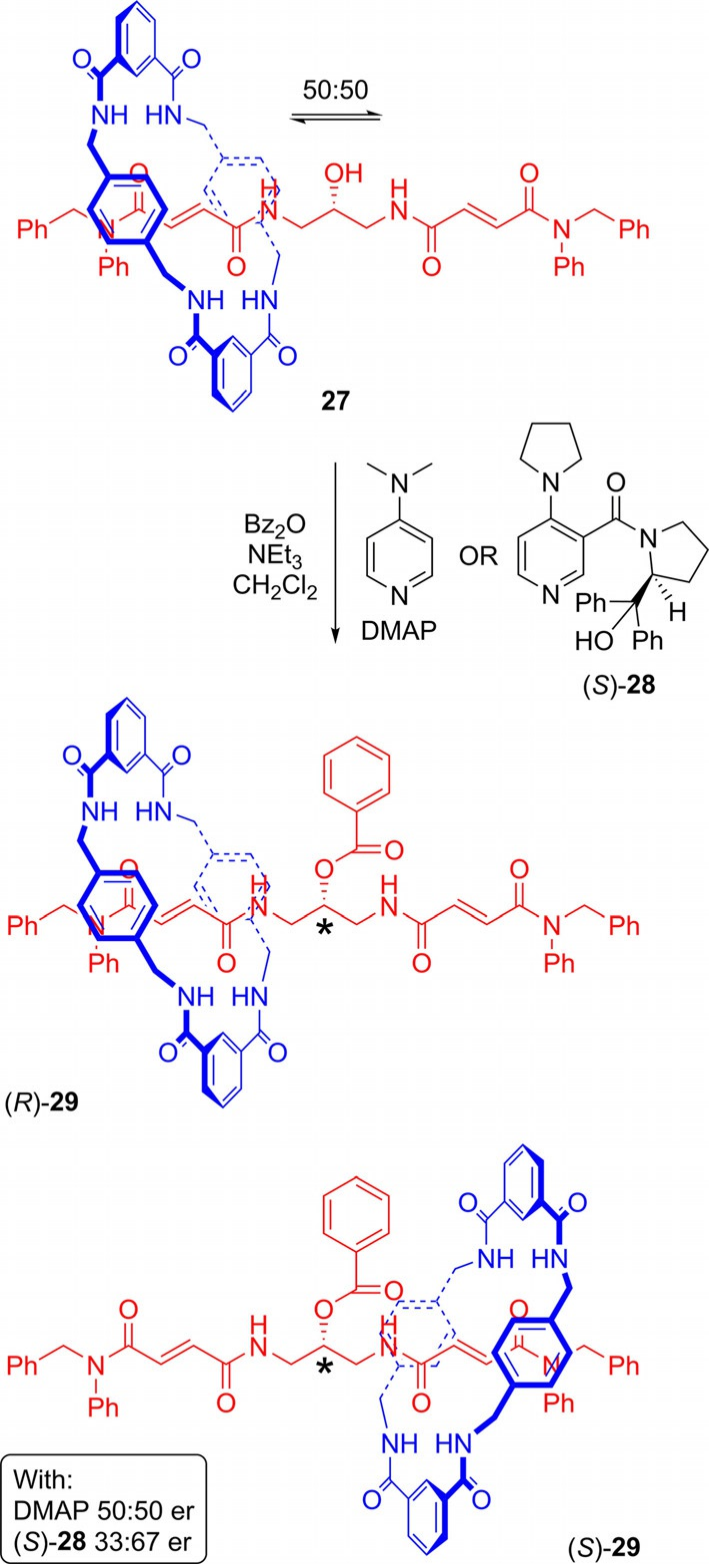
Leigh's conversion of achiral rotaxane **27** into rotaxane **29** that possesses point mechanical chirality. The asterisks mark the stereocentre in rotaxane **29**, which may be assigned according to standard CIP rules.

Mechanical chirality may arise in rotaxanes—which is more appropriately termed point mechanical chirality—when a macrocycle is trapped on one side of what would be a prochiral centre of the non‐interlocked axle component. This has been demonstrated in a rotaxane prepared by Leigh and co‐workers (Figure 17).^44^ At room temperature, the macrocycle of the achiral [2]rotaxane **27** can move between the two fumaramide functional groups on a symmetrical axle. However, if the alcohol at the centre of the axle is benzoylated, the macrocycle becomes trapped at one end of the axle, and the carbon atom attached to the benzoylated alcohol group becomes a stereogenic centre. If DMAP is used to catalyze the benzoylation reaction, a racemate of the point mechanically chiral rotaxane **29** is generated. However, use of a chiral catalyst allows for enantioselectivity, with rotaxane **29** being isolated with an enantiomeric ratio of 67:33 (*S*:*R*).

## 4 Chiral Catenanes and Rotaxanes in Application

Mechanically interlocked molecules are increasingly being put towards some form of functional application.^14^ For example, the three dimensional structures of certain catenanes and rotaxanes have been used to achieve selectivity in the binding and sensing of ionic and small molecular guest species.^15^ Meanwhile, others have been shown to act as catalysts, including examples for which controlled motion of the interlocked components is used to switch catalytic activity on and off.^16^ Examples of chiral catenanes and rotaxanes being used in such applications have been somewhat rare, but are now growing in number.

### 4.1 Chiral host–guest recognition

**Figure 18:**
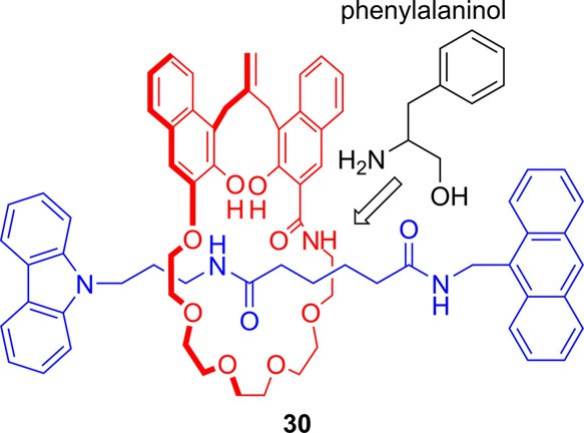
Structure of one enantiomer of Kameta and Hiratani's mechanically planar chiral rotaxane **30**, showing the tentative proposed mode of binding of phenylalaninol as suggested by the authors.

In 2006, Kameta and Hiratani reported upon the chiral sensing of phenylalaninol by a mechanically planar chiral rotaxane (Figure 18).^45^ The racemate of rotaxane **30** was prepared by a covalent bond formation (rather than template synthesis) approach.^46^ ^1^H NMR and fluorescence spectroscopic experiments provided evidence that l‐phenylalaninol was selectively bound through hydrogen bonding by one of the enantiomers of the rotaxane, and d‐phenylalaninol by the other in chloroform.

**Figure 19:**
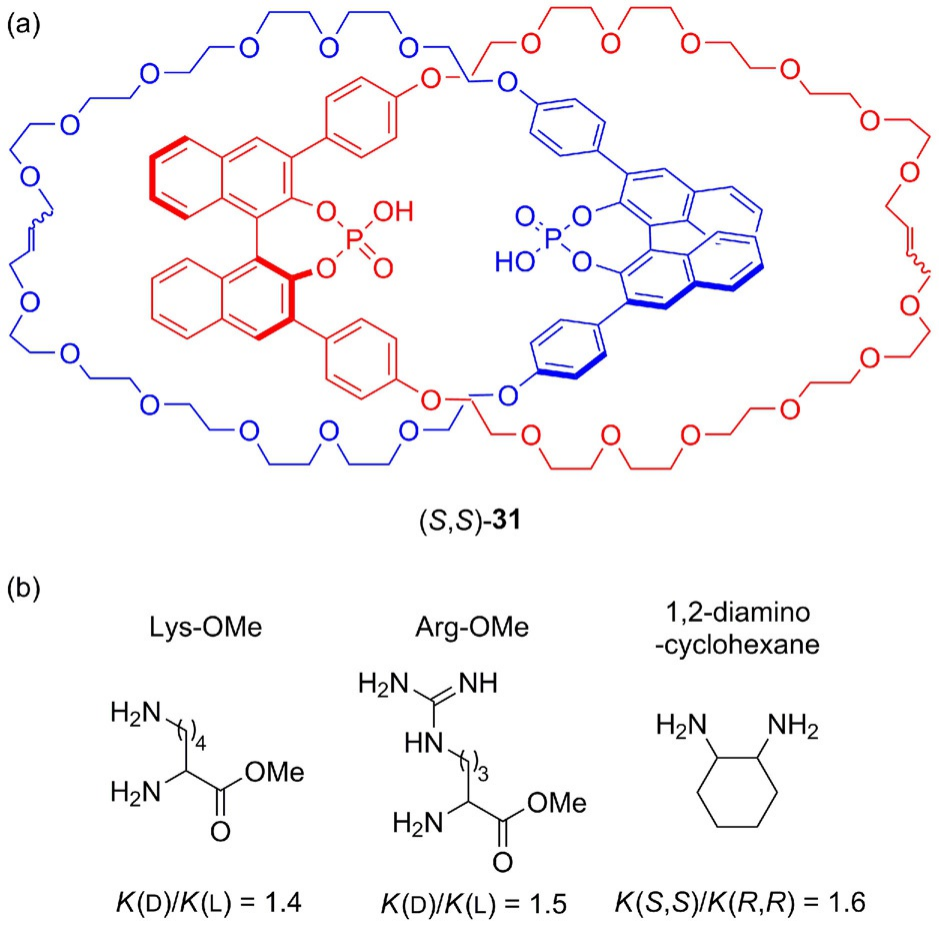
Niemeyers's catenane (*S*,*S*)‐**31** host prepared from enantiopure axially chiral binaphthyl‐phosphoric acid units: a) structure of catenane, and b) ratios of association constants for the binding of bis‐HCl salts of enantiopure diamine guests by the tetrabutylammonium salt of the catenane in [D_6_]‐DMSO.

Being studied as the racemate, the Kameta and Hiratani system is somewhat limited as an enantioselective host. To avoid this issue, Niemeyer and co‐workers prepared an enantiopure catenane by use of macrocyclic components containing an axially chiral binaphthyl‐phosphoric acid unit (Figure 19).^47^ Following removal of the calcium cation used to template the formation of the catenane, the researchers demonstrated that catenane **31** (as the bis‐tetrabutylammonium salt) possessed enantioselectivity for bis‐HCl salts of chiral diamine guests in DMSO. While the levels of enantioselective guest recognition were modest (*K*_fav_/*K*_disfav_=1.4–1.6), they were greater than for the non‐interlocked macrocycle.

**Figure 20:**
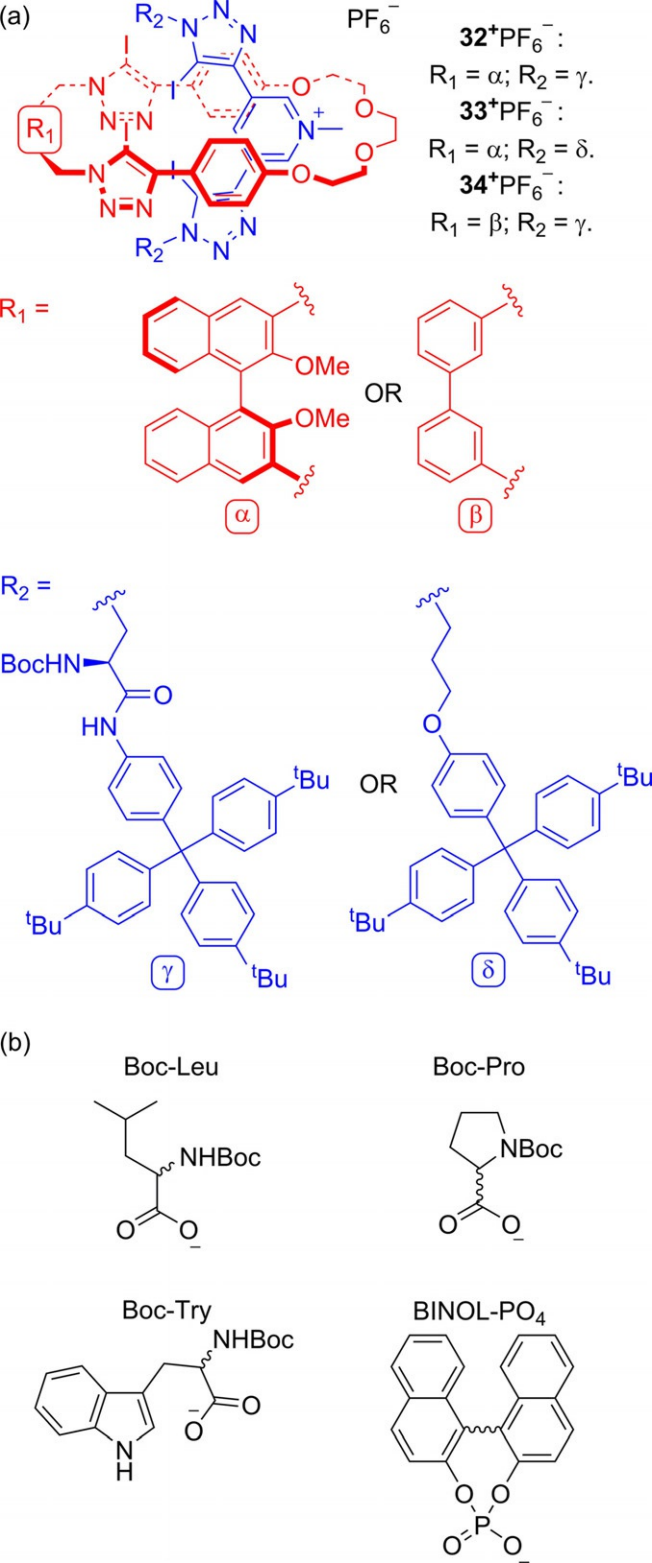
Beer's chiral halogen bonding rotaxanes **32**–**34^+^**PF_6_^−^ capable of enantioselective anion binding a) structures of rotaxanes and b) structures of chiral anions investigated.

Very recently, Beer and co‐workers have reported a detailed experimental and computational study of a set of chiral rotaxanes and their enantioselective anion binding properties (Figure 20).^48^ Prepared by active‐metal templating, both macrocycle and axle components possess iodotriazoles that can halogen bond to anionic guests in solution. By ^1^H NMR titrations, they demonstrated that rotaxanes **32** ^+^PF_6_^−^ and **33** ^+^PF_6_^−^ can bind chiral anions with enantioselectivities (*K*_fav_/*K*_disfav_) of up to 2.9 and 3.4 respectively (Table 1). Notably rotaxane **34** ^+^PF_6_^−^ exhibits negligible enantioselective behaviour towards any of the chiral anions investigated (*K*_fav_/*K*_disfav_ <1.2), meaning, for these rotaxanes, that the presence of a chiral macrocyclic component is essential to achieve reasonable levels of guest enantiodiscrimination.

**Table 1:** Ratios of association constants for the binding of enantiopure anionic guests (as tetrabutylammonium salts) by rotaxanes **32**–**34^+^**PF_6_^−^.

	Boc‐Leu	Boc‐Pro	Boc‐Try	BINOL‐PO_4_
**32** ^+^PF_6_^−[a]^	*K*_S_/*K*_R_=1.62	*K*_S_/*K*_R_=2.93	*K*_S_/*K*_R_=1.73	*K*_R_/*K*_S_=2.14
**33** ^+^PF_6_^−[b]^	*K*_R_/*K*_S_=3.41	*K*_R_/*K*_S_=1.50	*K*_R_/*K*_S_=1.61	*K*_R_/*K*_S_=1.78
**34** ^+^PF_6_^−[a]^	*K*_R_/*K*_S_=1.07	*K*_R_/*K*_S_=1.11	*K*_R_/*K*_S_=1.18	*K*_S_/*K*_R_=1.19

[a] In 98:2 [D_6_]‐acetone/D_2_O. [b] In 99:1 [D_6_]‐acetone/D_2_O.

### 4.2 Asymmetric catalysis

**Figure 21:**
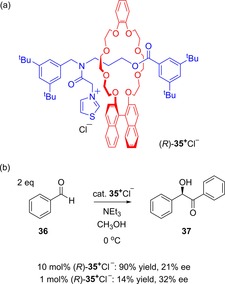
Takata's chiral rotaxanes for asymmetric benzoin condensations: a) example of one of the chiral rotaxanes studied, and b) example of benzoin condensation reaction where rotaxane (*R*)‐**35** ^+^Cl^−^ acted as a catalyst.

In 2004, Takata reported upon the use of rotaxanes possessing axially chiral binaphthyls to create a chiral environment to asymmetrically catalyze benzoin condensations (Figure 21).^49^ Good yields (up to 90 %), but with rather modest enantiomeric excesses (<32 % *ee*), were observed for rotaxanes, for which the chiral group was either part of the axle, or more impressively when the chiral group was part of the macrocycle (as depicted in Figure 21), thus transmitting the chiral information between the interlocked components to the thioazolium on the axle.

**Figure 22:**
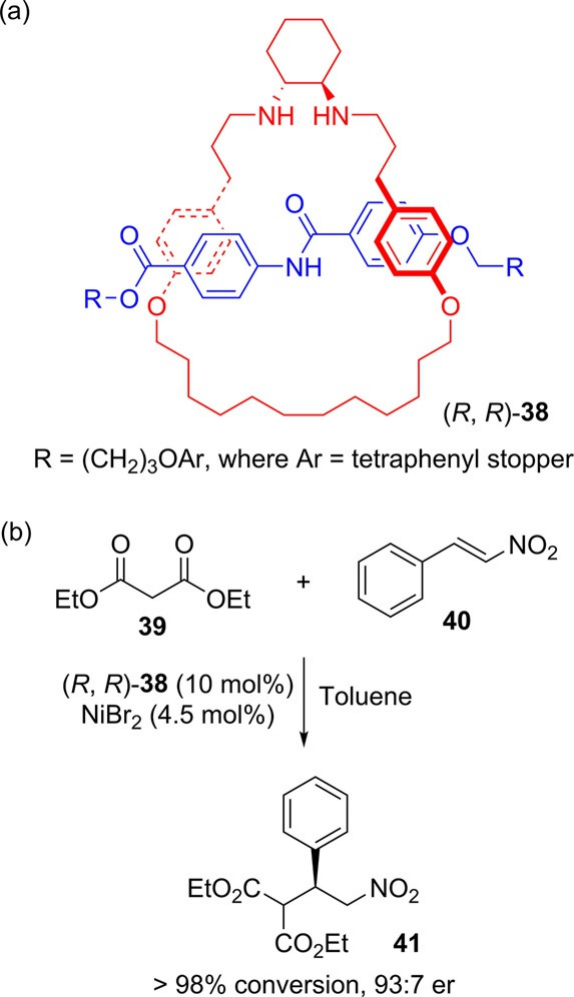
Leigh's chiral rotaxane for asymmetric transition metal catalysis: a) structure of rotaxane (*R*,*R*)‐**38**, and b) example of nickel‐catalysed reaction where rotaxane (*R*,*R*)‐**38** induces enantioselective bond formation.

Leigh and co‐workers have since reported on a number of chiral rotaxanes for asymmetric catalysis.^50^, ^51^, ^52^ For instance, an active metal template synthesized rotaxane possessing a chiral *C*_2_ symmetric *trans*‐cyclohexanediamine macrocycle, for use in enantioselective nickel‐catalyzed conjugate addition reactions (Figure 22).^50^ In comparison to an analogous acyclic ligand, rotaxane (*R*,*R*)‐**38** exhibited a much better enantiomeric ratio of product (93:7 compared to 68:32), but considerably slower reaction times (27 vs. 2 days for full conversion as determined by ^1^H NMR spectroscopy). These observations are consistent with the rotaxane improving expression of chirality arising from the two stereogenic carbon atoms (by reducing degrees of freedom), but also restricting access to the cation (since it is buried within the rotaxane structure while coordinated to the nitrogen amine atoms), thus reducing the rate of reaction.

**Figure 23:**
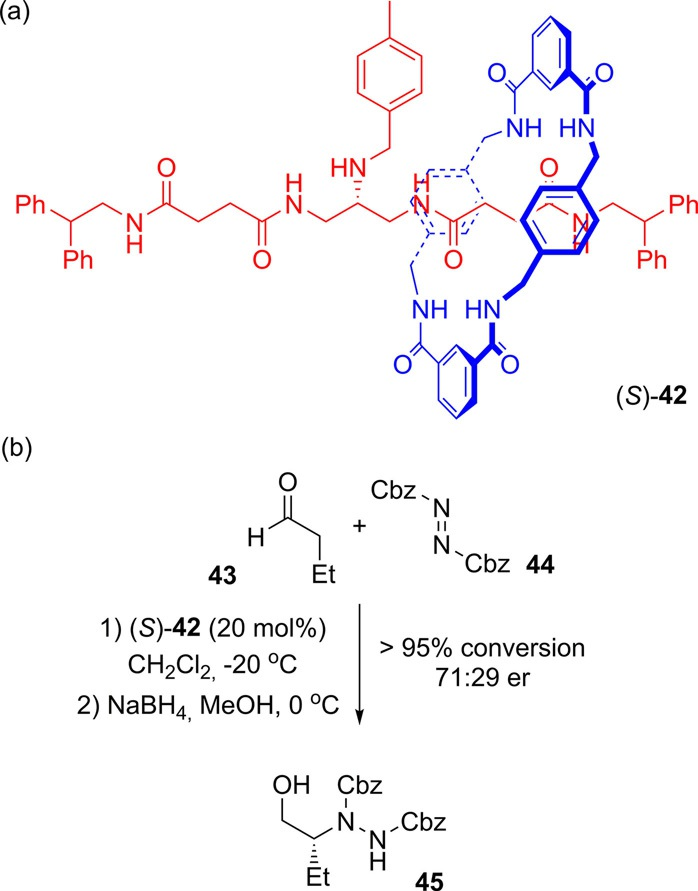
Leigh's point mechanically chiral rotaxane for asymmetric organocatalysis: a) structure of rotaxane (*S*)‐**42**, and b) example of enamine reaction where rotaxane (*S*)‐**42** induces enantioselective bond formation.

The same group has also exploited a point mechanical chiral rotaxane (*S*)‐**42** in catalysis (Figure 23).^51^ A secondary amine on the axle component may participate in enantioselective Michael addition and enamine reactions. The recorded enantiomeric ratio of products was somewhat low (68:32 and 71:29 were the best reported for the two types of reaction)—this is partly due to rotaxane (*S*)‐**42** being prepared in only 84 % *ee*—but the proof of principle was clearly demonstrated (enantiomeric ratios of 50:50 being observed in all cases where the achiral axle was used in place of the rotaxane).

**Figure 24:**
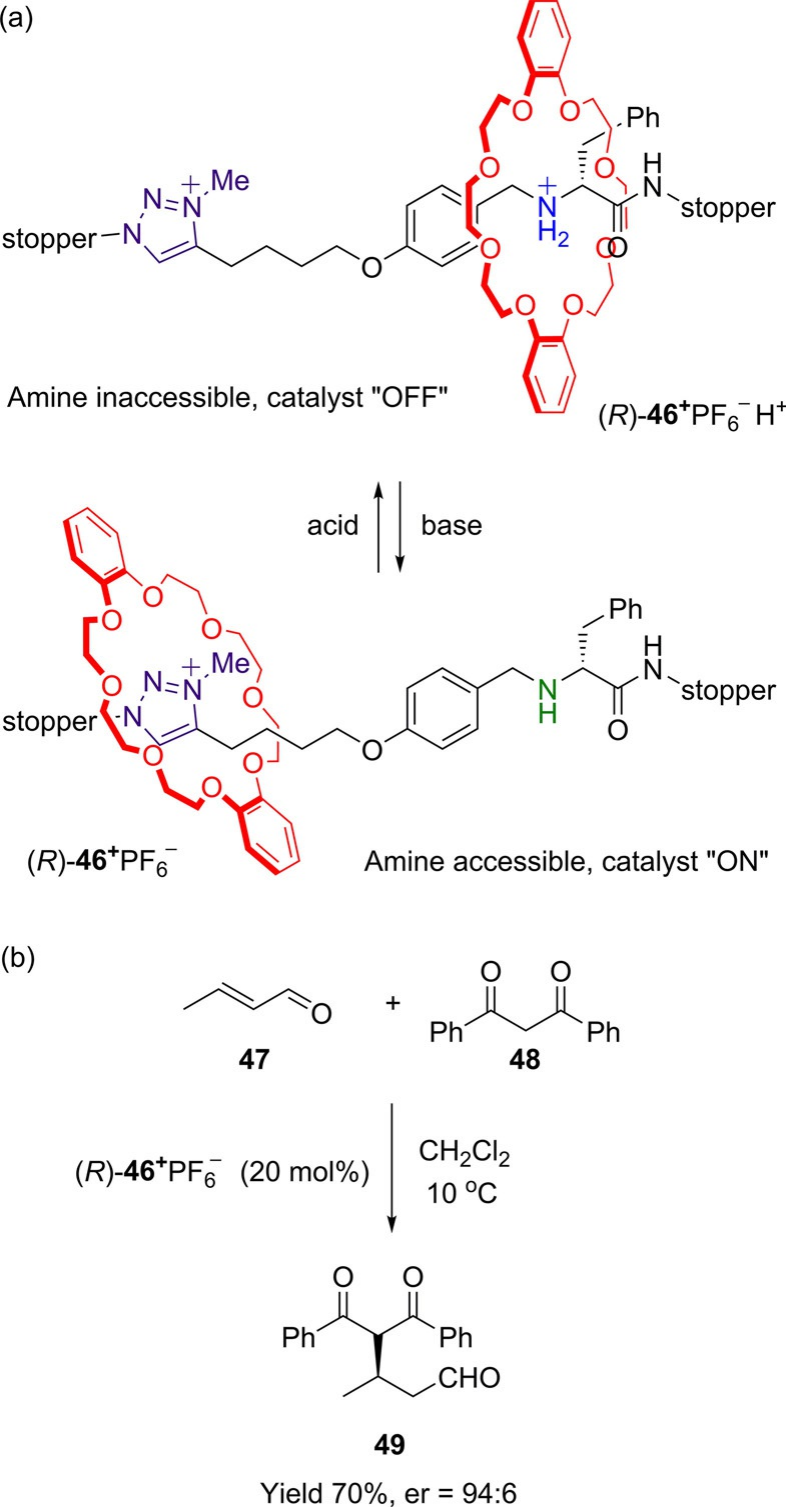
Leigh's switchable rotaxane for controlled asymmetric Michael additions: a) structure of rotaxane (*R*)‐**46** ^+^PF_6_^−^, and b) example of Michael addition asymmetrically catalysed by deprotonated rotaxane (*R*)‐**46** ^+^PF_6_^−^.

The researchers have also demonstrated that a switchable rotaxane may asymmetrically catalyze reactions in a controlled fashion (Figure 24).^52^ Rotaxane (*R*)‐**46** ^+^PF_6_^−^ includes two stations: in acidic conditions, the central secondary amine of the axle is protonated and the crown ether macrocycle resides over the resulting ammonium group, preventing the substrate from accessing the N atom. Once the amine is deprotonated, the crown ether moves to an alternative methyl triazolium station; substrates can then access the amine (which is bonded to a stereogenic carbon atom) and catalytic reactivity is thus turned on. This process may be reversed by addition of acid to reprotonate the amine, leading to translation of the macrocycle back over the reformed ammonium group. In its deprotonated state, rotaxane (*R*)‐**46** ^+^PF_6_^−^ can catalyze asymmetric Michael additions with reasonable conversion (60–70 %) and high enantiomeric ratios of products (up to 94:6).

**Figure 25:**
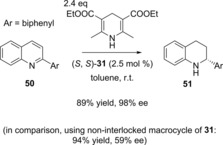
Example of transfer hydrogenation reaction catalyzed by catenane (*S*,*S*)‐**31** investigated by Niemeyer and co‐workers.

Very recently, Niemeyer and co‐workers have reported upon the use of binaphthyl‐phosphoric acid catenane (*S*,*S*)‐**31** (depicted in Figure 19) as a catalyst in asymmetric transfer hydrogenation reactions (Figure 25).^53^ Impressively, using catenane (*S*,*S*)‐**31** as the catalyst led to dramatically increased stereoselectivity compared to using the non‐interlocked macrocycle, with comparable yields (although notably slower reaction times). Detailed computational calculations provide evidence that the high stereoselectivities observed with the catenane are a direct result of its interlocked nature.

## 5 Conclusions

Future investigations into the preparation and study of chiral catenanes and rotaxanes have great potential. As highlighted in the Introduction, much progress has been made in developing new synthetic methodologies to prepare interlocked molecules since the early work on chiral catenanes and rotaxanes by the groups of Sauvage, Stoddart and Vögtle. Recent demonstrations of the application of chiral interlocked molecules in host–guest recognition and catalysis provide significant encouragement for researchers to work towards overcoming unresolved challenges in the field such as directly accessing enantiopure mechanically chiral catenanes and rotaxanes through enantioselective synthesis. By overcoming such challenges, we can look forward to realizing the full potential of interlocked molecules as useful three‐dimensional scaffolds in real‐world chiral applications.

## Conflict of interest

The authors declare no conflict of interest.

## Biographical Information

*Nick Evans graduated from Wadham College, University of Oxford with a First Class Masters in Chemistry (2006), before obtaining a DPhil in Inorganic Chemistry (2011), having worked on anion‐sensing rotaxanes and catenanes in the group of Prof. Paul Beer. After undertaking postdoctoral research on chiral lanthanide complexes with Prof. David Parker (Durham University), he took up a Lectureship in Chemistry at Lancaster University in 2013. His current research interests are in the area of functional supramolecular chemistry, including the synthesis and study of chiral interlocked host molecules for enantioselective guest recognition*.